# Pausing before verb production is associated with mild cognitive impairment in Parkinson’s disease

**DOI:** 10.3389/fnhum.2023.1102024

**Published:** 2023-04-11

**Authors:** Eduardo Inacio Nascimento Andrade, Christina Manxhari, Kara M. Smith

**Affiliations:** ^1^Department of Neurology, University of Massachusetts Chan Medical School, Worcester, MA, United States; ^2^NeuroNexus Institute, University of Massachusetts Chan Medical School, Worcester, MA, United States

**Keywords:** Parkinson’s disease, speech markers, cognition, language, MCI (mild cognitive impairment), verb

## Abstract

**Background:**

Cognitive dysfunction and communication impairment are common and disabling symptoms in Parkinson’s Disease (PD). Action verb deficits occur in PD, but it remains unclear if these impairments are related to motor system dysfunction and/or cognitive decline. The objective of our study was to evaluate relative contributions of cognitive and motor dysfunction to action verb production in naturalistic speech of patients with PD. We proposed that pausing before action-related language is associated with cognitive dysfunction and may serve as a marker of mild cognitive impairment in PD.

**Method:**

Participants with PD (*n* = 92) were asked to describe the Cookie Theft picture. Speech files were transcribed, segmented into utterances, and verbs classified as action or non-action (auxiliary). We measured silent pauses before verbs and before utterances containing verbs of different classes. Cognitive assessment included Montreal Cognitive Assessment (MoCA) and neuropsychological tests to categorize PD participants as normal cognition (PD-NC) or mild cognitive impairment (PD-MCI) based on Movement Disorders Society (MDS) Task Force Tier II criteria. Motor symptoms were assessed using MDS-UPDRS. We performed Wilcoxon rank sum tests to identify differences in pausing between PD-NC and PD-MCI. Logistic regression models using PD-MCI as dependent variables were used to evaluate the association between pause variables and cognitive status.

**Results:**

Participants with PD-MCI demonstrated more pausing before and within utterances compared to PD-NC, and the duration of these pauses were correlated with MoCA but not motor severity (MDS-UPDRS). Logistic regression models demonstrated that pauses before action utterances were associated with PD-MCI status, whereas pauses before non-action utterances were not significantly associated with cognitive diagnosis.

**Conclusion:**

We characterized pausing patterns in spontaneous speech in PD-MCI, including analysis of pause location with respect to verb class. We identified associations between cognitive status and pausing before utterances containing action verbs. Evaluation of verb-related pauses may be developed into a potentially powerful speech marker tool to detect early cognitive decline in PD and better understand linguistic dysfunction in PD.

## Introduction

Parkinson’s disease (PD) is characterized by both motor and non-motor symptoms, many of which relate to degeneration of the dopamine system and consequent basal ganglia dysfunction. While motor symptoms such as slowness of movements and muscle rigidity are well recognized, non-motor symptoms such as cognitive impairment as well as speech and language disorders also have a substantial impact on quality of life (Smith and Caplan, 2018). The language deficits in PD are often overlooked clinically, and mechanisms underlying these deficits have not been well elucidated.

One aspect language impairment in PD is action verb use. Studies have consistently found individuals with PD perform worse than controls on action verb production tasks including semantic tasks (lexical decision, similarity judgement (Fernandino et al., 2013a) and sentence processing (Fernandino et al., 2013b)), as well as naming (Cotelli et al., 2007) and generation of action verbs (Crescentini et al., 2008; Péran et al., 2009; Bocanegra et al., 2015, 2017). Action language comprehension as well as description and interpretation of observed actions is also affected (Boulenger et al., 2008; Humphries et al., 2016; Garcia et al., 2018). One hypothesis explaining these action verb deficits in PD is the embodied cognition theory (ECT). The ECT proposes that language, and in particular semantic, functions are scaffolded on underlying sensory-motor processes. Thereby, producing or understanding an utterance about a motor action would, at least in part, utilize brain networks required for execution of that action. In PD, the basal ganglia dysfunction that causes motor symptoms may lead to impairments in action-related language. Recent revisions of ECT include specific premotor “mirror” neurons (see Gallese and Cuccio for a review of these theories (Gallese and Cuccio, 2018)), which are also disrupted in PD. One caveat to evaluating the accuracy of the ECT is the fact that action verbs may be cognitively challenging as well (Alegre, et al., 2011; Aiello et al., 2022). While not directly in conflict of ECT and related theories, the cognitive challenge associated with verb production warrants further exploration in PD due to the well-established deficits in this condition. Verbs in general may be more cognitively complex than other parts of speech. Compared with nouns, verbs tend to be less concrete and their formulation and meaning is dependent on syntax. Further work is needed to clarify the importance of semantic representation, grammatical roles in sentences, lexical functions, and morphological structure for different classes of verbs in PD (Tyler et al., 2004). Since most prior research protocols have used constrained, non-naturalistic experimental tasks, such as reading tasks and naming, it is possible that confounding cognitive-linguistic demands have not been thoroughly accounted for. It also remains unclear how verb deficits manifest in real-life, daily speech in people living with PD. We therefore sought to explore motor and cognitive associations with action verb production in a spontaneous speech task in individuals with PD.

We assessed spontaneous speech production in PD participants with mild cognitive impairment (PD-MCI) and normal cognition (PD-NC). We evaluated pauses during speech as a quantitative measure of spontaneous action verb production deficits. Speech pauses are more frequent and longer in PD speakers compared to controls, and this occurs both before words within an utterance and between utterances (Ash et al., 2012; Smith et al., 2018; Lee et al., 2019; Whitfield and Gravelin, 2019). Pauses before words are thought to represent lexical retrieval time, while pauses before utterances represent semantic and syntactic planning and processing, as well as narrative organization (Rochester, 1973). We therefore proposed that pauses directly preceding action verbs and pauses before utterances containing action verbs (action utterances) might elucidate clinically relevant deficits in action and verb-related language production. We aimed to evaluate relative contributions of cognitive and motor dysfunction to action-related language production. Specifically, we compared pausing measures between participants with PD-NC and PD-MCI to characterize the linguistic planning challenges occurring in these conditions during a naturalistic speech task. We hypothesized that PD participants with cognitive impairment would demonstrate increased pausing before action verbs and utterances related to action verbs when compared to PD patients with normal cognition. This study is the first to our knowledge to assess spontaneous production of action verbs in speakers with PD in a context that approximates daily speech. We propose action language pauses as a potentially valuable speech marker of cognitive function in PD.

## Methods

### Participants

Participants were enrolled at the University of Pennsylvania Perelman School of Medicine and the University of Massachusetts Chan Medical School. Participants with idiopathic PD based on U.K. Brain Bank criteria (Hughes et al., 1993) were enrolled at University of Pennsylvania (*n* = 30) from 2013–2015 and at University of Massachusetts Chan Medical School (*n* = 62) from 2016–2022. PD participants were excluded if English was not their primary language, if they had undergone deep brain stimulation surgery, or had a history of other voice or laryngeal disorders.

All subjects completed an informed consent procedure in accordance with the Declaration of Helsinki and approved by the institutional review board of the local institution. At the University of Massachusetts, the protocol number was H00011523, approved 12/2/2016. At the University of Pennsylvania, this data was obtained under protocol number 842873 (University of Pennsylvania Centralized Observational Research Repository on Neurodegenerative Disease (UNICORN)).

### Assessment of cognitive, motor and language function

Motor severity was assessed using the Movement disorders society Unified PD rating scale (MDS-UPDRS, total Part III). MDS-UPDRS, total Part III axial score was available for a subset of 43 participants. Cognitive function was assessed using the Montreal cognitive assessment (MoCA) in all 92 participants. Participants enrolled after 2020 additionally underwent a battery of neuropsychological tests recommended by the MDS Task Force to determine a diagnosis of PD-MCI by level II criteria (Goetz et al., 2008; Dalrymple-Alford et al., 2010; Geurtsen et al., 2014). The cognitive battery includes the following tasks: Trail-making test A & B; Symbol digit modalities test; Boston Naming Test (30 item odd); Animal naming; Letter-guided verbal fluency; Judgement of line orientation (15 item odd); Boston Diagnostic Aphasia Examination; Hopkins verbal learning test (HVLT) – R immediate and HVLT-R Delayed and Recognition; Letter number sequencing; Brief visual memory test (BVMT) – R and BVMT-R Delayed and Recognition; Logical memory I (WMS-R, Anna Thompson story) and Logical memory II. Each case was reviewed by a panel of at least 3 clinicians to determine the consensus cognitive diagnosis of either mild cognitive impairment (MCI) or normal cognition (NC). Participants with dementia and those with indeterminate diagnosis were excluded. Cognitive diagnosis was available in 66 of 92 PD participants.

To assess spontaneous production of action-related language, participants underwent audio recording of a picture description task. Participants were asked to describe the Cookie Theft Picture from the Boston Diagnostic Aphasia examination (Kaplan and Weintraub, 1983). For this task, the participant was instructed that they would be given 60 s to describe the picture presented to them to the best of their ability, aiming to fill the entirety of the 60 s with their description. The Cookie Theft picture (Kaplan and Weintraub, 1983) task was selected because it leads to generation of natural speech, has been applied broadly in many neurological disorders and has been shown to provide a reasonable proxy of longer narrative tasks (Ash et al., 2013). Recording was performed using a hand-held digital recorder with a built-in unidirectional head-mounted microphone (Zoom H4n Pro Handy Recorder and Shure WH20 headset) at sampling rate of 44,100 Hz and 16 bits. The audio recordings were saved as. WAV files and analyzed using the software Praat (Paul and Weenink, 2011). The participant’s description of the Cookie Theft picture was then manually transcribed and segmented into utterances. Utterances were defined as one independent clause and all clauses or phrases dependent on it (Hunt, 1965). Fragments and segments were assessed to determine whether they related to the utterance immediately before or after, in terms of subject and informational content. If so, they were considered part of the utterance to which they referred (previous or following). If not, they were considered a separate utterance for the purposes of total utterance count but were not counted as action or non-action utterances.

**Figure 1:**
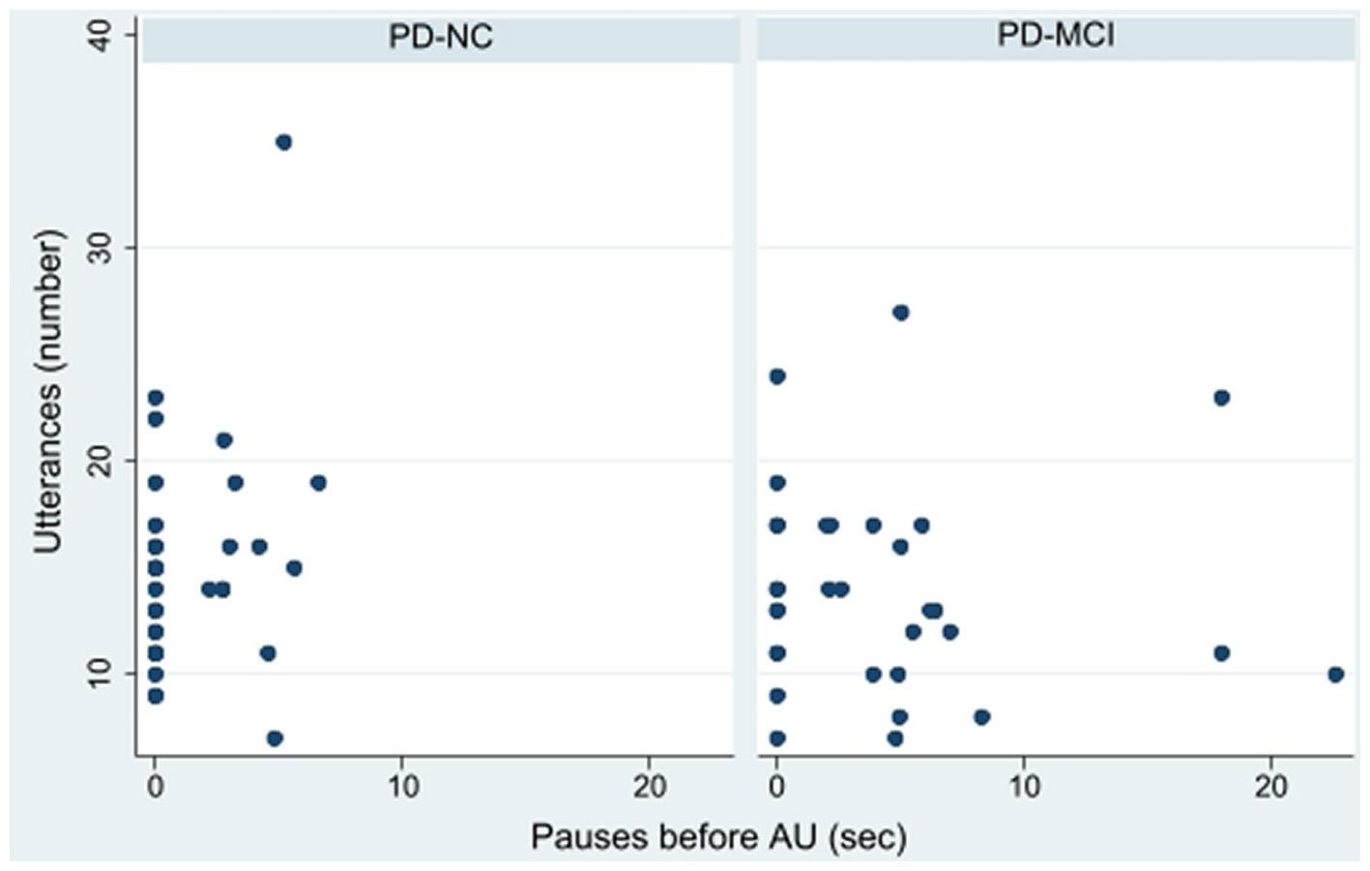
Action utterance pauses by cognitive status.

Verbs were manually identified and classified as non-action or action verbs. Auxiliary or linking verbs as well as modal verbs were classified as non-action verbs. These verbs help or modify another verb, for instance in forming tenses and the passive voice. Verbs relating to the speaker’s interpretation of the picture (for example, “I wonder” or “I think”) were classified as non-action verbs. Other verbs were classified as action verbs, and mostly described a motor action or intentional state (for example, “he wants” or “she is looking,” also see Supplementary Table S1 and Figure 1). Action utterances (AU) were defined as an utterance containing an action verb, and the remainder of the utterances were classified as non-action utterances (non-AU). Since our primary objective was to assess the cognitive-linguistic demand of utterance planning, utterances that contained one action verb and one non-action verb were considered an action utterance because the content is centered on an action.

Two raters (C.M., E.A.) performed the initial verb analysis and classification, and then 20% of randomly selected transcripts were independently analyzed by a second rater blinded to first rater’s scores (K.M.S.). Calculations for inter-rater consistency found only 8% of verb classification differed by rater.

Pauses were manually identified and measured using Praat. We measured each pause duration and rounded to the nearest 500 milliseconds. For instance, pauses between 500 and 1,000 milliseconds were rounded to 500 milliseconds, and pauses 1,000–1,500 milliseconds were rounded to 1,000 milliseconds. We selected these pause thresholds to better capture longer duration pauses thought to reflect cognitive-linguistic processes rather than speech motor processes (Whitfield and Gravelin, 2019). In accordance with Whitfield and Gravelin, we selected a shorter threshold (500 ms) for within-utterance pauses and a longer threshold (2000 ms) for between-utterance pauses. These lengths also ensured focus on clinically relevant pauses that would impact daily speech (Goldman-Eisler, 1972) and are within the range of pause thresholds that have been used in previous studies that assessed linguistic deficits in PD (Tjaden and Wilding Gregory, 2004; Goberman et al., 2005; Tjaden et al., 2014). We manually assessed the location of pauses within the transcripts and summed the number and duration of pauses proceeding AV, non-AV, AU and non-AU (see Supplementary Figure S1 for an example). The summed duration of pauses before each linguistic component was used to calculate a pause percent, corrected to account for differences in speech output and content between participants. The pauses before AU and non-AU were divided by the total number of utterances in the transcript. The pauses before AV and non-AV were divided by the total number of AV and non-AV. To control for variability in speaking rate, a modified words per minute variable was used (modified wpm = words divided by time after subtracting the pausing time). We applied the modified wpm variable as a measure of speech motor function and a proxy for articulatory rate.

### Statistical methods

The primary outcome was the corrected AU pause duration and secondary outcome was corrected AV pause durations. Visual plots demonstrated that these variables were left-skewed and not normally distributed, so non-parametric statistical methods were applied. We evaluated differences in these primary outcomes as well as other clinical and linguistic characteristics in the PD-NC group compared to the PD-MCI group using Wilcoxon rank sum tests. Within the total PD group, we used Spearman’s rho to assess correlations between pausing and linguistic variables with global cognitive function (MoCA) and with motor function (MDS-UPDRS Part III total and axial scores). To determine the association between cognitive status (PD-MCI vs. PD-NC) and our primary outcomes, we performed logistic regression, controlling for age, sex, motor severity (MDS-UPDRS Part III total). To assess whether action-related language was specifically predictive of PD-MCI status compared with non-action language, we compared logistic regression models with AU and non-AU as dependent variables. To explore underlying associations of specific cognitive domains with AU pausing performance, we assessed correlations with individual neuropsychological tests. All statistical tests were two-sided with significance set at *p* < 0.05. Analyses were performed in Stata (StataCorp. 2021. Stata Statistical Software: Release 17. College Station, TX: StataCorp LLC.).

## Results

### Baseline characteristics

Clinical characteristics are shown in Table 1. Those with PD-NC (*n* = 34) scored significantly higher on MoCA than those with PD-MCI (*n* = 32). Other baseline characteristics such as age, motor severity (MDS-UPRDS Part III) and education did not differ significantly between PD-NC and PD-MCI. Table 1 also provides the summary statistics of the linguistic markers for the overall PD group and by cognitive status. In terms of verb production performance, there was a numerical but non-significant decrease in action verb count in the PD-MCI group compared to PD-NC group. There were no significant differences in utterance, total word, and total verb counts between those with PD-NC and PD-MCI, while those with PD-MCI had a significantly lower speaking rate on the modified wpm measure than those with PD-NC (Table 1).

**Table 1:** Clinical and linguistic characteristics in the overall PD cohort and by cognitive status.

Variable	PD overall	PD-NC (median, IQR)	PD-MCI (median, IQR)	P (NC vs. MCI)
	(median, IQR)	N = 34	N = 32	
	N = 92			
Age (yrs)	67.0 (62.0–72.0)	66.5 (60–71.3)	67.0 (62.5–72)	0.74
Sex (# M, F)	45, 47	15, 19	16, 16	0.63
Education (yrs)	16.0 (15.0–18.0)	17.5 (16.0–18.0)	16.0 (13.5–18.0)	0.06
PD duration (yrs)	5.0 (3.0–10.0)	5 (2–8)	4 (4–10)	0.65
MoCA	27.0 (25.0–29.0)	28 (27–30)	26 (24.5–28)	0.0017
MDS-UPDRS III	26 (19–33)	21.5 (15–27)	27.0 (21.5–35.5)	0.99
MDS-UPDRS III axial	5 (3–6)	4 (2.5–5.5)	5.5 (4.0–7.0)	0.07
Words	143.5 (110.5–170.5)	163.0 (131–184)	128.5 (101–161.5)	0.02
Utterances	14 (11–17)	14.5 (11–16)	13.0 (10.5–17)	0.36
Verbs	24 (18.5–31.0)	26.0 (22–31)	24.0 (17.5–27.5)	0.14
Action verbs	12.5 (9–16)	14.0 (11–17)	12.5 (9–15.5)	0.14
Auxillary verbs	11 (8–15)	12.0 (9–16)	10.5 (7.5–13.0)	0.27
Pauses between utterances, sec	4.56 (0–9.53)	1.01 (0–6.25)	5.15 (0–15.05)	0.02
Pauses within utterances, sec	5.0 (3.0–7.8)	5.25 (3.5–7.5)	5.75 (2.75–9.0)	0.04
AU pauses	0.0 (0.0–4.95)	0 (0–2.75)	3.25 (0–5.68)	0.01
AV pauses	0.03 (0.0–0.08)	0.5 (0–1.0)	0.5 (0–1.0)	0.27
Wpm modified	143.48 (124.22–161.28)	155.35 (133.06–170.07)	138.93 (107.27–157.37)	0.02

MoCA, Montreal cognitive assessment; MDS-UPDRS III, Movement Disorders Society Unified Parkinson’s disease rating scale Motor Examination Part III. For all variables, the median/IQR reported is for the total duration of pause time before each linguistic feature, while the Wilcoxon rank sum value of p calculates the difference between the adjusted measurements as follows: Duration of pauses between utterances divided by total number utterances, Duration of pauses within utterances divided by total words, Duration of pauses before action or non-action utterances divided by total number utterances, duration of pauses before action verbs or non-action words divided by total number words. Modified wpm = words/(speaking time- total pause time).

### Association of cognitive and motor symptoms with action-related language pausing in PD

In the overall PD group, pauses before AU, non-AU and between all utterances were significantly correlated with MoCA (Table 2). Within-utterance pause duration was also significantly but less strongly correlated with MoCA, and this correlation applied to non-action verbs but not action verbs. Motor severity (MDS-UPDRS III) was not significantly correlated with any pause or linguistic measures. The axial score of the MDS-UPDRS Part III was significantly correlated only with the duration of within-utterance pauses, but not speaking rate (modified wpm). No individual neuropsychological tests correlated significantly with AU pauses (data not shown).

**Table 2:** Spearman correlations between pause measures for action/non-action language and cognitive and motor scores.

	MoCA (rho coefficient) *N* = 92	MDS-UPDRS Part III *N* = 92	MDS-UPDRS Axial score *N* = 43
**Pauses between utterances** ^a^	−0.46 *p* < 0.0001	0.05 *p* = 0.66	0.09 *p* = 0.58
AU pause^a^	−0.34 *p* < 0.001	0.09 *p* = 0.41	0.18 *p* = 0.25
Non-AU Pause^a^	−0.39 *p* = 0.0001	0.02 *p* = 0.89	−0.21 *p* = 0.17
**Pauses within utterances** ^b^	−0.24 *p* = 0.02	0.06 *p* = 0.58	0.47 *p* = 0.002
AV pause^b^	−0.05 *p* = 0.66	−0.04 *p* = 0.72	−0.02 *p* = 0.91
Non-AV pause^b^	−0.49 *p* < 0.0001	0.04 *p* = 0.70	0.07 *p* = 0.66
WPM^c^	0.38 *p* = 0.0002	−0.09 *p* = 0.37	−0.25 *p* = 0.11

a Corrected for the total number of utterances.

b Corrected for the total number of words, action verbs or non-action words as appropriate.

c Words/(time – between-utterance pause duration).

Several pause measures differed significantly between the PD-NC and PD-MCI groups. Participants with PD-MCI exhibited significantly longer total pause duration both *between* and *within* utterances than participants with PD-NC (Table 1). Those with PD-MCI also paused for significantly longer before AU than those with PD-NC (Figure 1). Comparing PD-MCI and PD-NC, there was no significant difference in pause duration before non-action utterances (rank sum *z* = −1.06, *p* = 0.29).

Logistic regression models revealed that AU but not non-AU pauses were significantly associated with cognitive diagnosis (Table 3), after adjusting for age, sex, and MDS-UPDRS III. For each additional 1.0 s of pausing before AU, the odds of PD-MCI increased by 32% (95% CI 5–66%, see Table 3).

**Table 3:** Logistic regressions using pause measures to predict PD-MCI status.

Variable	OR	95% CI	*p*
Pauses within utterances (all)^a^	1.17	1.01–1.35	0.032
AV pauses^b^	0.90	0.52–1.58	0.72
Pauses between utterances (all)^c^	1.13	1.03–1.25	0.013
AU pauses^c^	1.32	1.05–1.66	0.019
Non-AU pauses^c^	1.09	0.99–1.21	0.088

All models controlled for age, sex and motor severity (MDS-UPDRS Part III).

a Additionally controlled for total number words.

b Additionally controlled for total number action verbs.

c Additionally controlled for total number utterances.

## Discussion

We evaluated speech pauses as a novel measure of action verb and action-related language production in PD. By measuring pauses in a spontaneous speech task, we were able to describe naturalistic production of action verbs and utterances. We identified associations between these pause measures and cognitive but not motor impairment. Pauses before action utterances were significantly predictive of PD-MCI status independent of other motor and linguistic features.

Globally, pause measures between all types of utterances and within utterances were correlated with cognitive function but not motor impairment. Our results are consistent with prior studies in showing that pauses between utterances are associated with cognitive function (Ash et al., 2012). Pauses between utterances are thought to represent difficulty planning language. These pauses tend to be of longer duration than pauses between words or wicautionthin words in both PD and controls (Whitfield and Gravelin, 2019). We found that pauses between utterances were associated with cognitive status, and this occurred for both action and non-action related utterances. Therefore, our findings suggest there is difficulty planning and preparing for utterances involving all classes of verbs in PD. Further research is needed to explore pausing patterns in spontaneous utterances of different categories and degrees of lexical, semantic and syntactic complexity.

Pausing within utterances is thought to represent difficulty with lexical retrieval as well as speech motor processes. Prior work has found that within-utterance pauses are increased in PD (Rosen et al., 2006; Huber et al., 2012; Rusz et al., 2018; Whitfield and Gravelin, 2019). Arbitrary cut-offs have been used to define shorter duration “motor” pauses and longer duration pauses reflecting lexical retrieval. Analytical assessment including even very short (>15 ms) pauses, logarithmically transformed and subjected to Gaussian modeling, has revealed data-driven categorization of silences as articulatory (around <100 ms) and longer pauses rooted in lexical retrieval, prosodic and/or syntactic purposes (Rosen et al., 2010; Whitfield and Gravelin, 2019). Others have used a threshold of 250 ms, similar to our methodology, to discriminate pauses with lexical/cognitive-linguistic function within utterances (Goldman-Eisler, 1961; Boomer, 1965; Zeches and Yorkston, 1995; Ahn et al., 2014). We found a significant increase in within-utterance pauses in PD-MCI compared to PD-NC, which is consistent with the literature that pauses of this duration within utterances reflect cognitive decline in PD. Our results are notable because this association is present even in earlier cognitive decline (PD-MCI) while other studies have assessed those with more severe cognitive impairment (Ash et al., 2012). It has been proposed that basal ganglia dysfunction in PD contributes to both speech motor and cognitive/linguistic pauses through different circuits. One study assessing PD participants with deep brain stimulation of the subthalamic nucleus (STN DBS) found that stimulation altered pausing location and duration (Ahn et al., 2014). Further work remains necessary to understand the mechanisms underlying pausing abnormalities in PD as well as the role of therapies that improve basal ganglia function on pausing in speech.

Our results add to the growing literature aimed at understanding the mechanisms of action verb impairment in PD. Our study is the first to our knowledge to use pause measures to assess spontaneous production of different types of verbs and their context within utterances. This approach is more quantitative than measuring counts or number correct on linguistic tasks used in prior studies, and may be more reflective of real-time cognitive-linguistic processing demands. While we were able to identify associations between certain types of verbs and cognitive status, further work is needed to more precisely determine which verb properties most impact this association. We included verbs with different levels of action in our category of action verbs, while the non-action verb category included auxiliary and linking verbs. Our rationale for this approach was that additional verb sub-categorizations may be more subjective and difficult to replicate, and would not be appropriately supported by the picture description task stimulus. Nonetheless, this study contributes to the expanding conceptualization of action verb deficits in PD. There are many possible, but not mutually exclusive, explanations for action verb deficits in PD. One of the possible reasons is syntactically complexity, which we were unable to address with the constraints of this study. Lee et al. (2019) suggests that pausing at syntactic boundaries is related to syntactical complexity. Future work should determine if utterances containing action verbs are more syntactically complex than non-action utterances and if there is support for a specific impairment in action verb production after adjusting for this variable. Another hypothesized theory for action verb deficits in PD is the ECT and related theoretical frameworks. According to the ECT, the circuits responsible for motor actions contributes in part to produce language related to that action. Although, our work was not performed to refute or support the ECT, we found that action verb impairment is not correlated with general or axial motor scores suggesting that a simple connection may not be apparent in PD. More work is needed looking at action language in greater detail. For instance, high motion content action verbs have been found to be more impaired than lower motion content verbs in PD (Herrera et al., 2012; Bocanegra et al., 2017; Roberts et al., 2017; Speed et al., 2017), The concomitant influence of cognitive status was assessed in one study, in which action picture naming deficits were identified in PD participants both with and without cognitive impairment. Those without cognitive impairment had more selective difficulty with action verbs with higher motion content, whereas those with cognitive impairment had more widespread difficulties with all action verbs as well as manipulable nouns. However, cognitive status was determined by MoCA only (Bocanegra et al., 2017). In our study, the Cookie theft picture did not stimulate sufficient high motion content verbs to explore these associations. We instead found that pausing before action utterances may be a useful marker of PD-MCI status, and warrants further exploration and development. This finding is in line with another study identifying action verb comprehension in naturalistic language passages as a marker of cognitive impairment in PD subjects (Garcia et al., 2018). The mechanisms of cognitive impairment in PD are not fully understood but relate to a combination of widespread cerebral dopaminergic deficit, damage to sympathetic systems and to basal forebrain cholinergic systems (Aarsland et al., 2021). It is likely that patients with PD are heterogeneous in terms of which pathological changes contribute to cognitive decline within an individual. Understanding the specificity of linguistic deficits in PD, including action-related language impairments, could help elucidate these complex mechanisms and lead to individual-level characterization of cognitive systems.

Strengths of our study include cognitive categorization by MDS Tier 2 criteria for PD-MCI rather than MoCA alone, an approach that is more rigorous than prior studies. We also considered measures of speech motor and axial motor impairment, which may be more relevant to motor pathways tied to speech and language. Prior studies have focused on global motor severity, however the MDS-UPDRS Part III total is heavily dominated by limb motor impairment. Our approach to quantitatively measuring pauses provides a potentially rich and highly detailed data source, and this could be applied to other areas of speech and language research in PD. Finally, the use of a well-validated picture description task enhanced the clinical relevance of our study. Our findings describe verb production in typical, daily speech and may lead to improved understanding of speech deficits and new therapeutic approaches for PD.

Limitations to our study include a manual approach to identity and measure pauses. This approach may be time-consuming and impractical for larger scale studies, but was implemented as a proof-of-concept approach. Rapidly advancing speech recognition and signal processing algorithms should make similar analyses feasible and scalable. These advances would also allow for evaluation of much shorter pause durations with more precision. The exclusion of shorter duration pauses could have impacted our results, but may also have mitigated confounding introduced by including pauses with speech motor basis (Whitfield and Gravelin, 2019). Inclusion of shorter pauses could have enabled detection of more subtle or relatively well-compensated cognitive-linguistic processing difficulties, thus our approach may have made it more difficult to find associations with cognition. We also did not account for the potential role of breath-related pauses, though the physiology that causes breath-related pauses in PD were present across utterances types (Huber and Darling, 2011), which decreases the chances that our results are driven by respiratory mechanisms. Syntactic complexity may also influence pausing at syntactic boundaries (Lee et al., 2019), however this study does not allow us to control for this variable. Further work is needed to delineate the independent impact of action verbs in experimental paradigms controlled for syntactic complexity. Due to these multifaceted influences on pausing, we focused on pauses between utterances which should be more representative of cognitive demand.

Regarding the selection of the cookie theft task, one potential limitation is the number of events and motor actions portrayed in the picture. Discourse research could be strengthened by better validation of stimulus tasks according to psychometric standards (Stark Brielle, 2019; Stark Brielle et al., 2021). Additional discourse-based tasks could then be developed and utilized with greater precision to stimulate discourse production that is more representative of experimental aims. Another limitation was our verb categorization. We included verbs specifying a motor action that one could perform and psychological or abstract verbs in a single category. We categorized action verbs broadly due to the constraints of the cookie theft task, and our findings should be interpreted with caution. Our findings can be considered a first exploratory step toward characterizing pauses as linguistic markers in PD. Future research should analyze verb production according to more stringent verb categories, including physical vs. psychological action, high vs. low motion content, and part of body utilized for the motor action.

Finally, it should be noted that cognitive impairment in PD is heterogeneous and may be impacted by subject-specific factors and comorbidities. We excluded participants with dementia in order to better evaluate the cognitive-linguistic challenge of verb production *via* pauses. Participants with PD dementia may exhibit impaired processing and comprehension of the task itself making pauses more difficult to interpret (Ash et al., 2017). Additionally, Alzheimer’s type neuropathology frequently co-occurs with PD dementia and may be an independent source of language-based deficits (Irwin et al., 2012; Walker et al., 2015). Future research could include participants with dementia and utilize neuroimaging and/or biomarker measures in addition to cognitive testing to more fully elucidate the mechanisms underlying both cognitive deficits and action language impairments in PD.

In summary, our study is the first to show that pauses before utterances containing action verbs in PD are associated with mild cognitive impairment. While a deficit in linguistic planning exists for different types of semantic classes, action-related language seems to be more strongly correlated with cognitive impairment in PD. Our results raise questions for future studies to help elucidate the biological basis of deficits in different classes of verbs in PD. Location-specific pause measures could be further developed into useful markers to help detect and monitor cognitive impairment in PD.

## Data availability statement

The raw data supporting the conclusions of this article will be made available by the authors, without undue reservation.

## Ethics statement

The studies involving human participants were reviewed and approved by University of Massachusetts Chan Medical School IRB. The patients/participants provided their written informed consent to participate in this study.

## Author contributions

CM enrolled and assessed participants, collected, processing, and organized data, and contributed to drafting of manuscript. EA analyzed data and was primarily responsible for drafting manuscript. KMS designed and conceived of the study and was involved in all aspects of the research, contributed to writing and revising manuscript. All authors contributed to the article and approved the submitted version.

## Funding

Research reported in this publication was supported by the National Institute on Deafness and Other Communication Disorders of the National Institutes of Health under Award Number K23DC016656 awarded to KMS. The content is solely the responsibility of the authors and does not necessarily represent the official views of the National Institutes of Health.

## Conflict of interest

The authors declare that the research was conducted in the absence of any commercial or financial relationships that could be construed as a potential conflict of interest.

## Publisher’s note

All claims expressed in this article are solely those of the authors and do not necessarily represent those of their affiliated organizations, or those of the publisher, the editors and the reviewers. Any product that may be evaluated in this article, or claim that may be made by its manufacturer, is not guaranteed or endorsed by the publisher.
