# Supplementary material for: Pausing before verb production is associated with mild cognitive impairment in Parkinson’s disease
Source: Front Hum Neurosci. 2023 Apr 11;17:1102024. doi: 10.3389/fnhum.2023.1102024 (PMC10126398; doi:10.3389/fnhum.2023.1102024)
Supplement: Supplementary file 1 [file Table_1.docx]

Supplementary Table 1. Verb classification

| Action verbs | Non-action verbs– auxiliary and linking verbs |
| --- | --- |
| Examples: washing, falling, warning, passing, drying, splashing, wearing, wiping, raiding, reaching, pushing, looking (at) | Examples: is, has, was, appears, seems, going to, have to, can, need to, should, might, sound, feel |
|  |  |
| “the water is **splashing** onto the floor” | “this **looks** like a picture” |
| “housewife is **washing** dishes” | “the mother **seems** clueless” |
| “the boy is about to **fall**” | “the woman **seems** to be the mother” |
| “the girl **wants** a cookie” | “he **has** taken a cookie in his hand” |
| “he’s **reaching** for another one” | “the top of the jar **is** off” |
