# Supplementary figures and images for: Pausing before verb production is associated with mild cognitive impairment in Parkinson’s disease
Source: Front Hum Neurosci. 2023 Apr 11;17:1102024. doi: 10.3389/fnhum.2023.1102024 (PMC10126398; doi:10.3389/fnhum.2023.1102024)

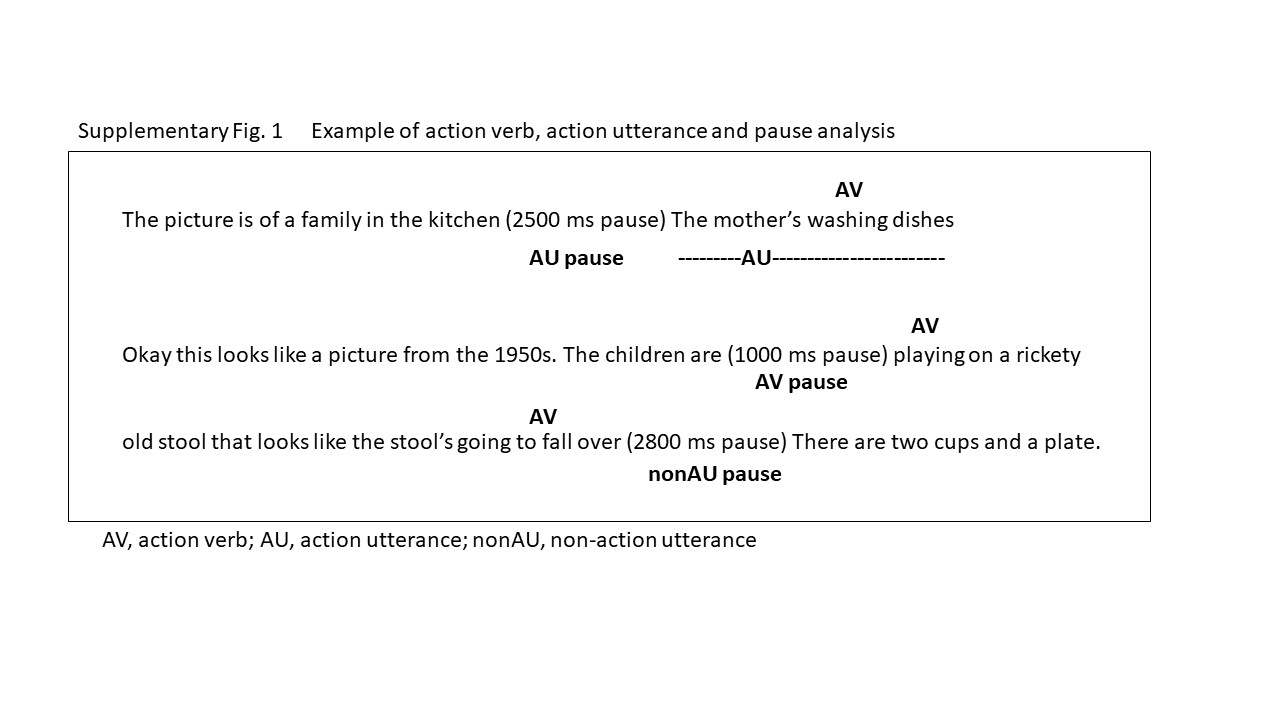

Supplement: Supplementary file 2 [file Image_1.JPEG]
